# Intraarticular use of tranexamic acid reduces blood loss and transfusion rate after primary total knee arthroplasty

**DOI:** 10.1186/s12891-019-2715-9

**Published:** 2019-07-27

**Authors:** Eric Tille, Jonas Mysliwietz, Franziska Beyer, Anne Postler, Jörg Lützner

**Affiliations:** University Center of Orthopaedics and Traumatology, University Medicine Carl Gustav Carus Dresden, Technische Universität Dresden, Fetscherstr. 74, 01307 Dresden, Germany

**Keywords:** Primary total knee arthroplasty, Total joint arthroplasty, Endoprosthetics, Blood loss, Transfusion rate, Tranexamic acid, Risk reduction, TXA, TKA

## Abstract

**Background:**

Tranexamic acid (TXA) is effective in reduction of hemorrhage after major surgical procedures. In total joint replacement it is commonly administered intravenously. Despite various studies regarding the safety of its antifibrinolytic effect there are contraindications for systemic use. In total knee arthroplasty (TKA) TXA can also be administered intraarticular. However, there is a lack of studies focusing on dosage, effectiveness and complications of this local treatment. This study aimed to evaluate if blood loss and transfusion rate can be reduced in primary TKA by local application of TXA.

**Methods:**

We included a total of 202 consecutive primary, unilateral TKA patients, 101 without and 101 with intraartricular application of 2 g TXA. Surgery was conducted after a standardized protocol. Blood loss, transfusion and complication rates were evaluated until three months after surgery. Blood loss was estimated using the hematocrit-value (Hk) prior and five days after surgery by Rosenecher’s and Mercuriali’s formula.

**Results:**

By the use of TXA a significant reduction of blood loss (Rosencher average 1220 ml vs 1900 ml, Mercuriali average 430 ml vs 700 ml *p* < 0,001) and transfusion rate (0% vs 24.75% of patients, p < 0,001) was observed. There were no differences regarding complication rates. Due to the lower cost of TXA compared to applied erythrocyte concentrates a side effect of the treatment was a cost reduction of € 1.609 within this cohort.

**Conclusions:**

The intraarticular application of 2 g TXA resulted in a significant reduction of blood loss and transfusion rate after primary TKA without increased complication rates. This method therefore seems to be a safe and cost effective instrument to reduce perioperative blood loss. However, it has to be considered that this is an off-label use.

## Background

With an increased life expectancy and a large incidence of osteoarthritis (OA) in older people the demand for TKA as ultimate treatment option for advanced knee OA will rise within the next 25 years [1]. Total knee arthroplasty is already one of the most frequent surgical procedures performed worldwide [2]. While the benefits of TKA are reduced pain and an improved function there are also risks, such as hemorrhage, thromboembolic events, infection and the need for additional surgical procedures. Especially older people with co-morbidities are at risk for complications. Due to hemorrhage up to one third receive blood transfusions which are associated with additional risks and costs [3–7]. Therefore, blood loss needs to be reduced.

Various approaches such as tourniquet, hypotensive anesthesia and various medical treatments have been evaluated to make TKA an even safer procedure. A very recent approach is the use of tranexamic acid (TXA). TXA is a hemostatic substance inhibiting the transformation of Plasminogen to Plasmin, therefore prohibiting fibrinolysis and reducing blood loss. While many studies have focused on the effectiveness of TXA reducing intra- and postoperative blood loss there is a need for further studies focusing on intraarticular administration of the substance and it’s dosage [8]. This study was performed to assess the effects of intraarticular TXA on peri- and postoperative blood loss and transfusion rate in unilateral, primary TKA. Within the current literature there seems to be no difference in the efficacy between 1.5 g and 3.0 g of intraarticular TXA in reducing perioperative blood loss during TKA [1]. Since higher dosages might increase the risk for adverse events, we chose a dosage of 2.0 g of TXA [9].

## Methods

After approval of the local ethics committee a total of 202 consecutive patients with knee OA scheduled for unilateral bicondylar TKA were included. No patients were excluded from analysis. The first 101 patients (control group) had surgery between July 2013 and February 2014 without TXA treatment. The next 101 patients operated on between March and October 2014 received 2 g (20 ml) of TXA intraarticular using a standard syringe after closure of the articular capsule. Otherwise the standardized procedure was not changed. In both groups a medial parapatellar approach, a tourniquet until implants were cemented and two intraarticular drainages were used. Tourniquets were inflated prior to the first skin incision and deflated before suture of the capsule. Within the treatment group these drainages were kept closed for a period of 2 h after surgery to ensure the effect of TXA.

The groups were compared for age, sex, BMI, comorbidities (ASA-score), preoperative anticoagulant medicines, laboratory parameters (hemoglobin (Hb), hematocrit (Hk), Quick, INR, aPTT), surgical parameters (duration of blood arrest, surgeon’s experience, duration of procedure) and adverse events (AE). AE’s were captured according to GCP by a study nurse until three months after surgery and categorized into surgical adverse events (superficial and deep infection, hematoma, nerve palsy, restricted range of motion, any re-operation), non-surgical adverse events (thromboembolic events, myocardial infarction, stroke, renal failure and all other adverse events which needed medical treatment).

Surgeries were performed according to comorbidity and preference of the patient under combined continuous peripheral nerve blocks with additional sedation, general anesthesia or spinal anesthesia. The tourniquet was inflated prior to the skin incision and deflated after cementing before closure of the capsule. The patella was not replaced. The experience of the surgeon was classified by the number of surgeries performed. A surgeon performing more than 50 TKA per year was considered experienced. A total of three experienced arthroplasty surgeons were present at all operations, in most cases leading the operation (Table 1). Postoperatively patients received an antithrombotic medication after a standardized protocol. The medication consisted of Rivaroxaban 10 mg once per day, a direct Anti-Xa-Antagonist (Xarelto) which was started 6-8 h after the surgical procedure. Patients with a preexisting anticoagulation received bridging as suggested by the angiologist, usually low-weight molecular heparine twice daily using half of the therapeutic dosage omitting the immediate preoperative dose. In presence of clinical symptoms (swelling, pain, positive Wells-Score) patients were further examined using ultrasonography.

**Table 1:** Preoperative and surgical data of control group vs. treatment group, given as mean (SD) and absolute (relative) frequencies

Analyzed criterion	Control group	Treatment group	*p*-value
Gender (% female)	65 (64.4%)	56 (55.4%)	0.196
Age [years]	68.2 ± 10,19	69.4 ± 10,1	0.396
BMI [kg/m^2^]	31.8 ± 6,78	31.46 ± 6,8	0.764
ASA-Score 1	2 (2%)	1 (1%)	0.394
2	49 (48.5%)	40 (39.6%)	
3	50 (49.5%)	59 (58.4%)	
4	0 (0%)	1 (1%)	
Cut-sew-time [min]	87.3 ± 16	87.9 ± 17	0.765
Tourniquet time [min]	68.1 ± 17	67.7 ± 21	0.890
Experienced surgeon	79 (%)	75 (%)	0.508
Diagnosis			
primary	90 (89.1%)	86 (85.1%)	0.254
posttraumatic	5 (4.9%)	11 (10.9%)	
inflammatory	6 (5.9%)	4 (3.9%)	
Kellgren-Lawrence			
°III	22 (21.8%)	6 (5.9%)	0.001
°IV	79 (78.2%)	95 (94.1%)	

The primary endpoint of the study was the blood loss. In order to estimate the total blood loss a mathematical approach was used. After calculation of the blood volume using Nadler’s formula the approximate blood loss was determined using Mercuriali’s and Rosechner’s formula [10–12]. This ensures that the total blood loss is captured.

Formula 1 – calculation of total blood loss after Mercuriali et al. [10, 11]

$$ blood\ loss= blood\ volume\ x\ \left({Hct}_{preop}-{Hct}_{5d- postop}\right)+{V}_{TE}\ \left[ ml\right] $$

Hct_preop_ = preoperative Hematocrit, Hct_5d-postop_ = Hematocrit on day 5 after surgery, V_TE_ = total transfused erythrocyte volume [ml].

Formula 2 – calculation following the OSTHEO forumla after Rosencher et al. [12]

$$ V\mathrm{TL}=\frac{V_{TBL}}{0.35} $$

$$ {V}_{TBL}={V}_{URL}+{V}_{CRL} $$

$$ {V}_{URL}={V}_{initial}-{V}_{final} $$

$$ {V}_{initial}=\mathrm{BV}\times {Hct}_{preop} $$

$$ {V}_{final}=\mathrm{BV}\times {Hct}_{postop} $$

$$ \mathrm{BV}=\mathrm{bs}\times \mathrm{cf} $$

$$ \mathrm{for}\ \mathrm{females},\mathrm{cf}=2430,\mathrm{for}\ \mathrm{males},\mathrm{cf}=2530 $$

$$ \mathrm{bs}\ \left[{\mathrm{m}}^2\right]=0.0235\times \mathrm{heigth}{\left[\mathrm{cm}\right]}^{0.42246}\times \mathrm{body}\ \mathrm{weigth}\ {\left[\mathrm{kg}\right]}^{0.51456} $$

V_TL_ = total blood loss [ml]; V_TBL_ = total red blood cell loss[ml], V_URL_ = uncompensated red blood cell loss [ml]; V_CRL_ = compensated red blood cell loss [ml] = combination of all the red blood cell’s from transfusion; V_initial_ = the red blood cell volume before surgery [ml]; v_final_ = the red blood cell volume after surgery [ml]; BV = Blood volume [ml]; bs = body surface [m^2^], cf = regulating variable relating to sex; blood volume is calculated after Nadler’s formula.

Secondary endpoints were transfusion rates, length of hospital stay, cost effectiveness and complication rate. Indications for blood transfusion based on a protocol of Steininger et al. were constant throughout the entire study period. Erythrocyte concentrates were administered if one of the following criteria was met [13, 14]:

- Hb < 7-8 g/dl, in patients with preexisting cardiac disease
- Hb < 6 g/dl, in patients without cardiac conditions
- presence of clinical symptoms of inadequate oxygen supply (i.e. dyspnea, tachypnea, reduced consciousness, etc.)

All data was collected prospectively and evaluated using the patient’s medical records, anesthesia protocols and the digital hospital information system ORBIS (AGFA Healthcare GmbH, Bonn, Germany).

To detect a clinically relevant difference of at least 200 ml blood loss between both groups with a power of 0.8 and significance level of 0.05 a minimum of 89 patients per group was necessary.

The software SPSS (release 23 for Windows, IBM, Armonk, New York, USA) was used for data analysis. Data description was based on means and standard deviation (SD) for continuous variables and absolute and relative frequencies for categorical variables. In not normally distributed values medians were used. Groups were compared using paired t-test for normally distributed continuous variables, Mann-Whitney-U-Test if not normally distributed and chi-square test for categorical variables. Significance level was set at *p* < 0.05.

## Results

Both groups were not different for demographic data, preoperative medication and surgery related data (Table 1).

Analysis of the preoperative hemostasis parameter, hemostasis influencing medication and calculated blood volume did reveal no difference between the two groups (Tables 2, 3). Only hemoglobin value was significantly different, but with a difference of only 0.48 g/dl mmol/l not clinically relevant.

**Table 2:** Preoperative hemostasis parameters

Parameter	Control group	Treatment group	Significance (p)
blood volume[l]^a^	4.98 ± 0.92	5.03 ± 0.88	0.730
Hematocrit (HKT)	0.4 ± 0.04	0.41 ± 0.03	0.149
Quick [%]	109.49 ± 89.16	94.42 ± 19.14	0.1
INR	1.01 ± 0.14	1.06 ± 0.21	0.052
apTT [s]	28.93 ± 3.86	28.92 ± 4.5	0.986
Hemoglobin [g/dl]	13.38 ± 1.34	13,86 ± 1,18	0.008

Given as mean (SD),^a^blood volume was calculated using Nadler’s formula [10]

**Table 3:** Preoperative medication with influence on hemostasis

Substance	Control Group (n)	Treatment Group (n)	*p* - value
PAI	9	9	1,000
NSAR	40	51	0,120
LMWH	14	18	0,459

PAI platelet aggregation inhibitor, e.g. Aspirin, Clopidogrel; NSAID non-steroid anti-inflammatory drugs, LMWH low molecular weight heparin

**Fig. 1:**
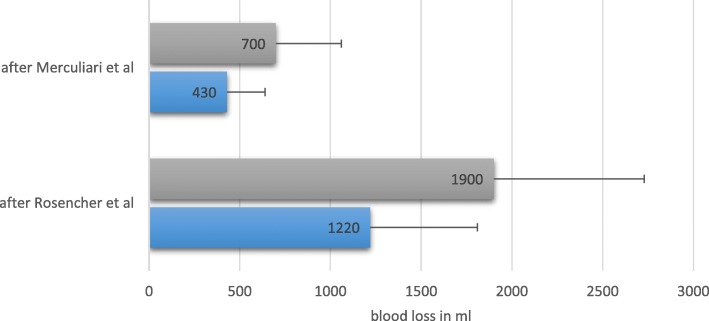
Perioperative blood loss calculated using Rosenchers and Merculiari’s formula. Blood loss in ml. Grey = control group, blue = treatment group

There was a highly significant reduction in blood loss in the TXA group calculated with Mercuriali’s (*p* < 0,001) and Rosencher’s formula (p < 0,001, see Fig. 1 for details).

In the control group 22 patients (21.78%) needed an erythrocyte transfusion (21 patients 2 concentrates, 1 patient 4 concentrates) while none in the treatment group needed a transfusion (p < 0,001). The average length of the hospital stay was not different between both groups. While patients of the control group spent an average of 8.3 days in the hospital, the mean hospital stay in the treatment group was 8.8 days. It needs to be considered that length of hospital stay in Germany depends mainly on organizational aspects (e.g. availability of rehabilitation) and not primarily on discharge criteria.

Adverse events were not different between control and treatment group. Overall seven deep vein thrombosis (3 vs 4) and one additional muscle vein thrombosis in the TXA group were recorded. There has been one proximal and three distal deep vein thrombosis within the treatment group. Within the control group three distal deep vein thrombosis were observed. Also within the control group we found one patient with a heart rhythm disorder (tachycardia) and one patient with a post-surgical hemorrhage due to thrombocytopenia in context of liver cirrhosis. Furthermore, manipulation under anesthesia were necessary twice in the TXA group due to restricted range of motion. No further adverse events were observed.

The cost of 2 g TXA was € 17.50. This resulted in additional treatment costs of € 1`767.50 in the TXA group. In the control group a total of 46 erythrocyte concentrates (each € 73.40) were administered, which resulted in additional costs of € 3`376.30. This accounts for an effective cost reduction of € 1`608.80 in the TXA group.

## Discussion

In this study the intraarticular use of TXA in primary TKA resulted in a significant reduction of blood loss and blood transfusions without increased complication rates.

Orthopedic surgeries in general are the procedures with the highest demand for blood transfusions [15]. They are estimated to account for up to 10% of total transfusions. Approximately 45% of patients with major surgical procedures require a transfusion due to extensive perioperative blood loss [16]. 39% of these transfusions are associated with joint replacement surgery [17]. Complications of blood transfusions are rare but can lead to severe consequences for the patient. Therefore, a reduction of blood loss and consequently of transfusion rates is desirable. Application of TXA seems to be an appropriate treatment to achieve this goal. Several studies have shown that TXA can reduce hemorrhage rate in orthopedic procedures [18].

TXA is usually given intravenously but this is contraindicated in patients with several comorbidities such as a history of thrombosis, myocardial infarction or severe renal dysfunction. Since patients undergoing TKA often suffer of severe comorbidities (in this study 54% ASA grade 3 and 4) not all patients can be treated with prophylactic intravenous TXA. Alternatively, intraarticular application has been described. Seo et al. showed that local administration is superior to intravenous treatment [19]. Therefore, a local approach, minimizing systemic side effects whilst reducing the risk of postoperative bleeding, seems preferable. This is consistent with the results of this study. Depending on the equation 2 g TXA saved 270 ml (Mercuriali’s formula) or 680 ml blood (Rosencher’s formula).

The results for blood loss calculated using Rosechner’s and Mercuriali’s formulas tend to be much higher than surgeons and anesthesiologists estimate. As Sehat et al. have proven, there is a hidden blood loss that nearly doubles the visible loss during and after surgery [7, 20]. This is due to blood loss into swabs und covers during the surgery and hemorrhage into the tissue after surgery has been finished. It is therefore necessary to favor formulas including also non-visible blood loss and observing a longer period of time and not only the surgery itself [21]. In order to include this invisible blood loss the equations of Mercuriali and Rosenecher have been developed. Their advantage is that they take the pre- and postoperative hemoglobin level into account. In this study an average reduction of blood loss by 40% accompanied by a reduction of blood transfusions within the treatment group could be observed. Studies of Alshydra et al. and Li R et al. have also investigated this issue and displayed similar results demonstrating that intraarticular TXA application reduces the transfusion rate in TKA and THA [3, 22].

Previous studies have demonstrated that drain clamping itself can result in a reduced blood loss. Cao et al. described an approximately 100 ml higher blood loss in the non-clamped group. Zan et al. observed an increase in drainage volume of 120 ml if not-clamped. Within our study only the drains in the treatment group were clamped in order to allow the TXA to be effective. While this may contribute to a lower blood loss within the treatment group the overall reduction in blood loss after application of TXA was much higher than the aforementioned approximately 100 ml [23, 24].

Prior study results, such as provided by Karam et al. or Aguilera et al., showed a reduction of transfusion rate after postoperative intravenous TXA application [25, 26]. However the safety of intravenous application of TXA, especially regarding the development of thromboembolic events, is still an issue [8]. In this study there was no difference in adverse events until 3 months after surgery especially focusing on thromboembolic events. This is consistent with the studies of Pinsornask et al. and Liu et al. which demonstrated that the local application of TXA does not lead to an increased complication rate in comparison to the intravenous application [27, 28]. Overall, the majority of the studies on the local use of TXA demonstrate no increase in complication rates [3, 22, 29–31].

While some studies in the past have reported a slight reduction in length of hospital time [3, 32] after TXA application, other studies did not find a reduction as in our study. [28, 33, 34]. Overall a comparison between these study results is difficult, since differing healthcare systems account for different overall conditions including the length of hospital stay.

As an additional effect, the use of TXA was cost saving. In a prior study Shander et al. have remarked the noticeable effect of blood transfusion upon total hospital cost ( [16]). Due to a high intraoperative and postoperative blood loss, orthopedic surgery does account for a high percentage of these transfusions. Consequently the use of TXA resulted in reduced hospital costs [35–37].

This study has some limitations. There was no randomization of the participants. However, evaluation of the demographic and preoperative data displayed no differences between the two groups. It can therefore be assumed that both groups are homogenous. The blood loss could not be measured directly but was calculated using two commonly used equations. Therefore, the real blood loss might be different. However, this method was applied to both groups and therefore the difference between both groups should be reliable. Although there was an identical standard for transfusions over the whole study period different physicians were involved and might have different judgements for the indication to blood transfusion.

## Conclusion

This study demonstrated that the intraarticular use of tranexamic acid in primary unilateral TKA did significantly reduce postoperative blood loss and consequently reduced the need for blood transfusions without an increase in adverse events, especially thromboembolic events. However, it needs to be considered that TXA is not approved for intraarticular use in some countries and might be an off-label use. This needs to be discussed with the patient.

## Data Availability

The datasets used and analysed during the current study are available from the corresponding author on reasonable request.

## Abbreviations

%: Percent
€: Euro
aPTT: partial thromboplastin time
ASA: American society of Anesthesiologists Score
Avg: average
BMI: Body mass Index
EK: Erythrocyte concentrate
g: grams
g/dl: gram per deciliter
Hb: Hemoglobin
Hk: Hematocrit
INR: International normalized ratio
kg/m^2^: Kilogram per Meter^2^
LMWH: low molecular weight heparin
mg/dl: Milligramm per deciliter
min: Minute
Ml: Milliliter
NSAR: non-steroidal antirheumatics
p: significance
PAI: platelet aggregation inhibitors
THA: total hip arthroplasty
TKA: Total knee arthroplasty
TXA: Tranexamic acid
vs: versus

## References

[1] Kurtz S, Ong K, Lau E, Mowat F, Halpern M (2007). Projections of primary and revision hip and knee arthroplasty in the United States from 2005 to 2030. J Bone Joint Surg Am.

[2] Bleß H.-H., Kip M. (2016). Weißbuch Gelenkersatz.

[3] Alshryda S, Mason J, Vaghela M (2013). Topical (intra-articular) tranexamic acid reduces blood loss and transfusion rates following total knee replacement: a randomized controlled trial (TRANX-K). J Bone Joint Surg Am.

[4] Good L, Peterson E. Lisander B. Tranexamic acid decreases external blood loss but not hidden blood loss in total knee replacement. 2003. 10.1093/bja/aeg. 10.1093/bja/aeg111 12697586

[5] Hiippala S, Strid L, Wennerstrand M (1995). Tranexamic acid (Cyklokapron) reduces perioperative blood loss associated with total knee arthroplasty. Br J Anaesth.

[6] Jansen A J, Andreica S, Claeys M, D'Haese J, Camu F, Jochmans K (1999). Use of tranexamic acid for an effective blood conservation strategy after total knee arthroplasty. British Journal of Anaesthesia.

[7] Sehat KR, Evans R, Newman JH (2000). How much blood is really lost in total knee arthroplasty? Correct blood loss management should take hidden loss into account.

[8] Fillingham YA, Ramkumar DB, Jevsevar DS (2018). The Safety of Tranexamic Acid in Total Joint Arthroplasty: A Direct Meta-Analysis. J Arthroplasty.

[9] Wong J, Abrishami A, El Beheiry H (2010). Topical application of tranexamic acid reduces postoperative blood loss in total knee arthroplasty: a randomized, controlled trial. J Bone Joint Surg Am.

[10] Nadler SB, Hidalgo JH, Bloch T (1962). Prediction of blood volume in normal human adults. Surgery..

[11] Mercuriali F, Inghilleri G (1996). Proposal of an algorithm to help the choice of the best transfusion strategy. Curr Med Res Opin.

[12] Rosencher N, Kerkkamp HEM, Macheras G (2003). Orthopedic surgery transfusion hemoglobin European overview (OSTHEO) study: blood management in elective knee and hip arthroplasty in Europe. Transfusion..

[13] Steiniger K (2015). Intra- und postoperative Blutverluste bei elektivem Hüftgelenksersatz in der Orthopädie: Analyse patienten- und eingriffsspezifischer Prädiktoren.

[14] Habler O, Meier J, Pape A, Zwißler B. Indikation zur Bluttransfusion bei orthopädischen Eingriffen. Orthopade. 2004;33(7). 10.1007/s00132-004-0672-x. 10.1007/s00132-004-0672-x 15138679

[15] Slover James, Lavery Jessica A., Schwarzkopf Ran, Iorio Richard, Bosco Joseph, Gold Heather T. (2017). Incidence and Risk Factors for Blood Transfusion in Total Joint Arthroplasty: Analysis of a Statewide Database. The Journal of Arthroplasty.

[16] Shander A, Hofmann A, Ozawa S, Theusinger OM, Gombotz H, Spahn DR (2010). Activity-based costs of blood transfusions in surgical patients at four hospitals. Transfusion..

[17] Stanworth SJ, Cockburn HAC, Boralessa H, Contreras M (2002). Which groups of patients are transfused? A study of red cell usage in London and Southeast England. Vox Sang.

[18] Fillingham YA, Ramkumar DB, Jevsevar DS (2018). The Efficacy of Tranexamic Acid in Total Knee Arthroplasty: A Network Meta-Analysis. J Arthroplasty.

[19] Seo J-G, Moon Y-W, Park S-H, Kim S-M, Ko K-R (2013). The comparative efficacies of intra-articular and IV tranexamic acid for reducing blood loss during total knee arthroplasty. Knee Surg Sports Traumatol Arthrosc.

[20] Sehat KR, Evans RL, Newman JH (2004). Hidden blood loss following hip and knee arthroplasty. Correct management of blood loss should take hidden loss into account. J Bone Joint Surg Br.

[21] Wallroth K (2013). Prospektiv randomisierte Studie zum Vergleich des Blutverlustes bei elektiver Implantation einer Knietotalendoprothese mit und ohne Blutsperre.

[22] Alshryda S, Sukeik M, Sarda P, Blenkinsopp J, Haddad FS, Mason JM (2014). A systematic review and meta-analysis of the topical administration of tranexamic acid in total hip and knee replacement. Bone Joint J.

[23] Cao JG, Wang L, Liu J (2015). The use of clamped drainage to reduce blood loss in total hip arthroplasty. J Orthop Surg Res.

[24] Zan P, Yao JJ, Fan L (2017). Efficacy of a Four-Hour Drainage Clamping Technique in the Reduction of Blood Loss Following Total Hip Arthroplasty: A Prospective Cohort Study. Med Sci Monit.

[25] Karam JA, Bloomfield MR, DiIorio TM, Irizarry AM, Sharkey PF (2014). Evaluation of the efficacy and safety of tranexamic acid for reducing blood loss in bilateral total knee arthroplasty. J Arthroplast.

[26] Aguilera X, Martinez-Zapata MJ, Bosch A (2013). Efficacy and safety of fibrin glue and tranexamic acid to prevent postoperative blood loss in total knee arthroplasty: a randomized controlled clinical trial. J Bone Joint Surg Am.

[27] Pinsornsak P, Rojanavijitkul S, Chumchuen S (2016). Peri-articular tranexamic acid injection in total knee arthroplasty: a randomized controlled trial. BMC Musculoskelet Disord.

[28] Liu Q, Geng P, Shi L, Wang Q, Wang P (2018). Tranexamic acid versus aminocaproic acid for blood management after total knee and total hip arthroplasty: A systematic review and meta-analysis. Int J Surg.

[29] Panteli M, Papakostidis C, Dahabreh Z, Giannoudis PV (2013). Topical tranexamic acid in total knee replacement: a systematic review and meta-analysis. Knee..

[30] Jang B, Kao M, Bohm MT, Harris IA, Chen DB, MacDessi SJ (2014). Intra-articular injection of tranexamic acid to reduce blood loss after total knee arthroplasty. J Orthop Surg (Hong Kong).

[31] Yu X, Li W, Xu P, Liu J, Qiu Y, Zhu Y (2015). Safety and efficacy of tranexamic acid in Total knee arthroplasty. Med Sci Monit.

[32] Chimento GF, Huff T, Ochsner JLJ, Meyer M, Brandner L, Babin S (2013). An evaluation of the use of topical tranexamic acid in total knee arthroplasty. J Arthroplast.

[33] Nielsen CS, Jans O, Orsnes T, Foss NB, Troelsen A, Husted H (2016). Combined intra-articular and intravenous tranexamic acid reduces blood loss in Total knee arthroplasty: a randomized, double-blind, placebo-controlled trial. J Bone Joint Surg Am.

[34] Georgiadis AG, Muh SJ, Silverton CD, Weir RM, Laker MW (2013). A prospective double-blind placebo controlled trial of topical tranexamic acid in total knee arthroplasty. J Arthroplast.

[35] Sepah YJ, Umer M, Ahmad T, Nasim F, Chaudhry MU, Umar M (2011). Use of tranexamic acid is a cost effective method in preventing blood loss during and after total knee replacement. J Orthop Surg Res.

[36] Gillette BP, Maradit Kremers H, Duncan CM (2013). Economic impact of tranexamic acid in healthy patients undergoing primary total hip and knee arthroplasty. J Arthroplast.

[37] Ralley FE, Berta D, Binns V, Howard J, Naudie DDR (2010). One intraoperative dose of tranexamic acid for patients having primary hip or knee arthroplasty. Clin Orthop Relat Res.

